# A Pulsed Thermographic Imaging System for Detection and Identification of Cotton Foreign Matter

**DOI:** 10.3390/s17030518

**Published:** 2017-03-04

**Authors:** Jesse Kuzy, Changying Li

**Affiliations:** 1 Institute for Artificial Intelligence, University of Georgia, Athens, GA 30602, USA; jesse.kuzy25@uga.edu; 2 College of Engineering, University of Georgia, Athens, GA 30602, USA

**Keywords:** cotton foreign matter, thermography, pulse-phase thermography

## Abstract

Detection of foreign matter in cleaned cotton is instrumental to accurately grading cotton quality, which in turn impacts the marketability of the cotton. Current grading systems return estimates of the amount of foreign matter present, but provide no information about the identity of the contaminants. This paper explores the use of pulsed thermographic analysis to detect and identify cotton foreign matter. The design and implementation of a pulsed thermographic analysis system is described. A sample set of 240 foreign matter and cotton lint samples were collected. Hand-crafted waveform features and frequency-domain features were extracted and analyzed for statistical significance. Classification was performed on these features using linear discriminant analysis and support vector machines. Using waveform features and support vector machine classifiers, detection of cotton foreign matter was performed with 99.17% accuracy. Using frequency-domain features and linear discriminant analysis, identification was performed with 90.00% accuracy. These results demonstrate that pulsed thermographic imaging analysis produces data which is of significant utility for the detection and identification of cotton foreign matter.

## 1. Introduction

During harvest and transportation, cotton is contaminated by foreign matter. The most common type of foreign matter is botanical matter from the cotton plants: leaf fragments, hulls, stems, seeds, seed coats, and so on; followed by foreign fibers and textiles made of cotton, plastic, and jute; and least common are inorganic debris and oily substances [1]. Prior to ginning, seeds and seed coats are also present. During baling and transportation, cotton may be contaminated with baling twine, fragments of module cover, or grease and oil from machinery. Exogenous debris such as windblown paper and plastic fragments are also occasionally incorporated during harvest. Much of this debris, especially large pieces of debris such as seeds and stems, is removed during the ginning and cleaning process. Other types of foreign matter may be broken into smaller fragments and not removed; leaf fragments are the most prevalent of these. Following cleaning, cotton is graded according to, among other metrics, its foreign matter content. Cotton containing a high proportion of foreign matter results in defects in textiles, as well as interfering with processing equipment by introducing oil and dust [2]. The detection of cotton foreign matter is therefore a paramount financial consideration for growers, ginners, and textile manufacturers.

The current industry standard device for cotton grading is the High-Volume Instrument (HVI), which measures properties of the cotton including fiber length, uniformity, and strength, micronaire, color, and foreign matter content [3]. This is frequently coupled with human grading, especially for additional analysis of the foreign matter content. It is notable that this system is not capable of determining the type of foreign matter present, nor are human graders tasked with making this determination. United States Department of Agriculture (USDA) standards describe the foreign matter content of cotton batches in terms of “leaf grade”. The use of this term implies that foreign matter is comprised primarily of leaves and similar botanical debris. Though this is generally the case, it masks the diversity of foreign matter types encountered in cotton lint. The Advanced Fiber Information System (AFIS), another industrial system, shares this shortcoming [4].

The low cost and ease of implementation of charge-coupled device (CCD) and complementary metal-oxide-semiconductor (CMOS) color cameras have led many researchers to attempt detection using red-green-blue (RGB) machine vision. Xu et al. used CCD cameras and Xenon illumination to show a strong correspondence between CCD, HVI, and human grading on estimates of foreign matter content and cotton color measurements [5]. Later studies by Yang et al. combined color and ultraviolet (UV) illumination for foreign matter detection and achieved a mean detection accuracy of 92.34% using both color and shape features [6,7]. However, white foreign matter was problematic, and other researchers have pointed out that shape characteristics are not ideal features, since mechanical harvesters can produce foreign matter scraps in an endless variety of shapes [8].

Significant research has been devoted to the identification of foreign matter by Fourier-Transform Near-Infrared Spectroscopy (FT-NIR). These techniques use the absorbance spectra of cotton and foreign matter as the basis for discrimination between substances. Fortier et al. have produced a pair of studies using this technique demonstrating 97% accuracy of classification on a set of four foreign matter types (hull, leaf, seed, stem) [9] and 98% accuracy on a set of eight foreign matter types (hull, leaf, seed coat, seed meat, stem, plastic, twine) [10]. The primary weakness of FT-NIR detection techniques is that they are point-based, presenting difficulties for high-volume application; another weakness is the necessity of compiling libraries of the spectral characteristics of the materials of interest, a significant complication [2].

A combination of machine vision methods and FT-NIR methods is hyperspectral imaging, which uses the transmittance or reflectance modes to collect spectra for every visible pixel of a sample. Jiang et al. showed that spectral features derived from this method provide a statistical basis to separate all of 15 foreign matter types except brown leaves and bract [11]. Guo et al. achieved some success in this area, particularly for the detection of foreign fibers, which are a common contaminant in Chinese cotton fields [12]. Using mean NIR spectra collected from a set of 16 foreign matter types and cleaned cotton lint, Zhang et al. achieved an accuracy of classification of 96.5% using linear discriminant analysis (LDA) classifiers, including 100% accuracy on the cotton lint [13].

Some researchers have taken advantage of the natural fluorescence of cotton foreign matter to perform detection. Gamble and Foulk [14] were able to reliably identify leaves and hull by fluorescence spectroscopy. Mustafic et al. replicated these findings using fluorescence imaging under blue illumination, and additionally demonstrated excellent classification of paper, plastic module cover, and commercial plastic bag under UV excitation [15]. Using X-ray microtomography, Pai et al. achieved an average classification rate of 96% on a sample set including seed coats, bark, and polypropylene [16]. However, the expense of the instrumentation and the necessity of strict controls on worker exposure to radiation are both obstacles to industrial implementation of this technology.

One imaging modality that has not been applied to the problem of cotton foreign matter detection is thermal imaging. Thermal imaging has been applied to a wide variety of post-harvest quality evaluation tasks, such as detecting mechanical damage and bruising in apples [17]; evaluating the quality of apple wax coatings [18]; detecting soft spots on tomatoes [19]; and detecting insect infestation in wheat kernels [20]. Pulsed and lock-in thermographic techniques have been used to detect apple bruises [21,22,23], with Varith et al. theorizing that the observed difference in the temperatures of bruised and healthy tissues can be attributed to different thermal diffusivities in the tissues. Specifically, higher thermal diffusivities in bruised tissues create a “thermal window” which allows the rapid flow of heat from the exterior to the interior of the apple, resulting in a lower surface temperature in bruised regions. Meinlschmidt and Maergner demonstrated that hazelnuts have different thermal properties from typical foreign bodies present in harvested nuts, and developed a thermal imaging system to detect these foreign bodies by heating the nuts and foreign bodies with a flash lamp [24]. Detection was successfully conducted and the researchers concluded that this was possible due to the differing thermal properties of the nuts and foreign bodies [25]. Since it is also the case that cotton and cotton contaminants differ in their thermal properties (although the degree of difference may be minimal for some contaminants), it should be possible to discriminate between cotton and its contaminants using similar techniques. Furthermore, since cotton is dried with hot air in early ginning stages and then returned to the general processing floor where temperatures are nearer to ambient, both cotton and the foreign matter it contains are already exposed to a sharp rise and fall in temperature as part of the ginning process. This may enable use of the technology without any exogenous heat source.

Some potential advantages of thermal sensing over fluorescence and hyperspectral imaging which motivate exploration into the use of thermal sensing include the reduced cost compared to hyperspectral imaging systems, and the ability to determine material properties of samples which do not show a strong fluorescence under ultraviolet stimulation.

The goal of this paper was to explore the feasibility of using a pulsed thermographic imaging technique to detect and classify the cotton foreign matter. Specific objectives were to (1) design and construct a pulsed thermographic analysis system with image acquisition software and a fully-integrated data processing pipeline; (2) extract and evaluate the effectiveness of frequency-domain features and thermal waveform features; and (3) perform classification of common cotton contaminants using these features.

### Pulse-Phase Thermography

Thermographic analysis is the estimation of a target’s temperature based on the magnitude of thermal radiation (infrared radiation) emitted by the target. The relationship between the emitted radiation and the temperature of the observed object is governed by the Stefan–Boltzmann law [26], Equation (1):

(1) $$E={\varepsilon \sigma T}^{4},$$

where E is the total emitted radiation in W/m^2^, ε is the emissivity of the object, dimensionless, σ is the Stefan–Boltzmann constant in W/m^2^·K^2^, and T is the absolute temperature in K. Since temperature is the dominating term on the right-hand side of the equation, and since emissivity is a static property for most substances, any observed changes in emitted radiation can be attributed to changes in the temperature of the object.

One method that can reveal additional properties of the target object is active thermography: in active thermographic techniques, an external source of heat is applied to the object. The change in the object’s temperature over time is then monitored. A specific technique within this family is pulsed thermography: a radiative heat source is turned on and off at set time intervals, applying one or more pulses of heat to the object. The redistribution of heat within the object is influenced by a variety of material properties, such as thermal diffusivity, heat capacity, and the geometry of the object. In addition to radiated energy, the object may lose heat from conduction or convection, both of which will also be related to various properties of the material.

**Figure 1:**
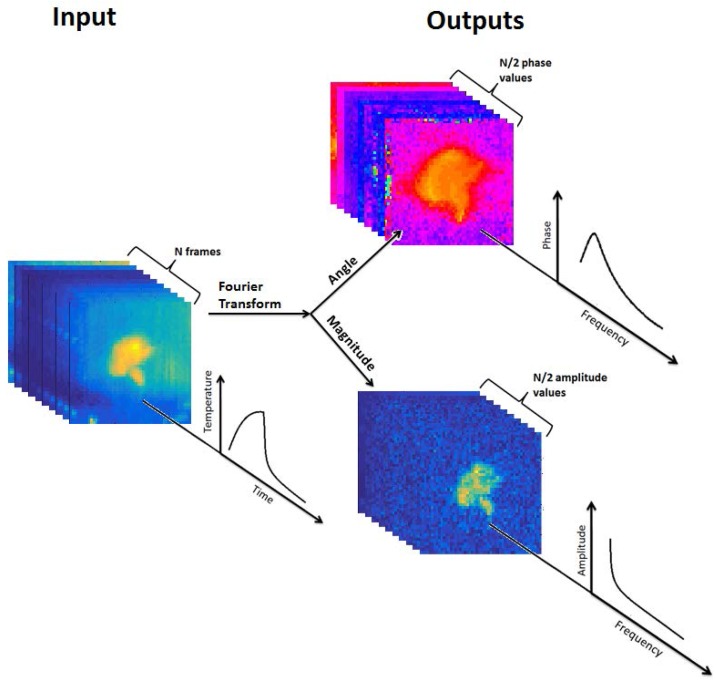
Illustration of temporal Fourier transform of cotton foreign matter pulsed thermography videos: temporal image stack produces phasegram and ampligram image stacks.

Pulse-phase thermography is an analytical technique for pulsed thermographic analysis [27]. In this technique, the changing temperature of each pixel of the observed object is considered as a thermal signal with a temporal dimension, and the Fourier transform is applied to this signal, decomposing it into a sum of sinusoidal components (Figure 1). Since the full characterization of the thermal signals of the object pixels is contained in both the amplitude and phase data, both of these values are of interest as potential features for discrimination of samples.

## 2. Materials and Methods

### 2.1. Samples

**Figure 2:**
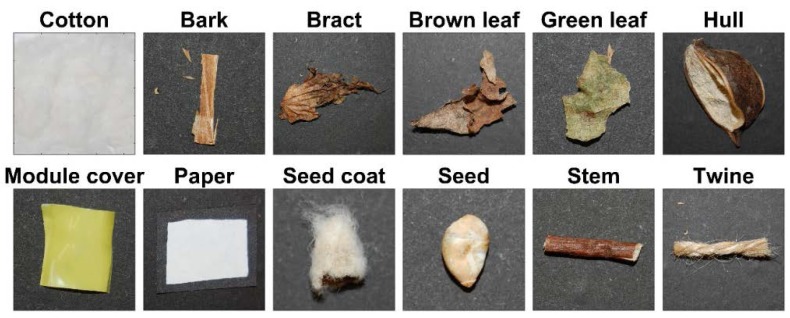
Color photographs of foreign matter and cotton lint samples representative of the classes used in this study.

For this study, eleven types of common cotton contaminants were examined: bark, bract, brown leaves, green leaves, hulls, module cover, paper, seed coats, seeds, stems, and twine (Figure 2). Botanical foreign matter samples were extracted from seed cotton samples of four cultivars planted and harvested in 2012: Delta Pine 0912; Delta Pine 1050; PhytoGen 499; and FiberMax 1944. Paper and twine samples were purchased through common consumer channels. Module cover samples were taken from a cotton gin in Tifton, GA, USA. Large scraps were collected and roughly cut into small squares with side lengths in the range 2–5 mm. For each foreign matter type, 20 samples were analyzed. Additionally, 20 samples of cleaned cotton lint of the aforementioned cultivars were analyzed. In total, 240 samples of cotton and foreign matter were used in this study.

Samples were relatively homogenous in their sizes within each class. It is known that contaminants show significant variance in size and geometry as encountered in cotton lint, but adjusting for these variations presents significant challenges, since controlling for edge effects is among the more problematic issues in thermal modeling. For this reason, a wide variety of geometries of samples was not employed in this study. This issue may be addressed in later studies.

### 2.2. Pulsed Thermographic Imaging System and Data Acquisition

**Figure 3:**
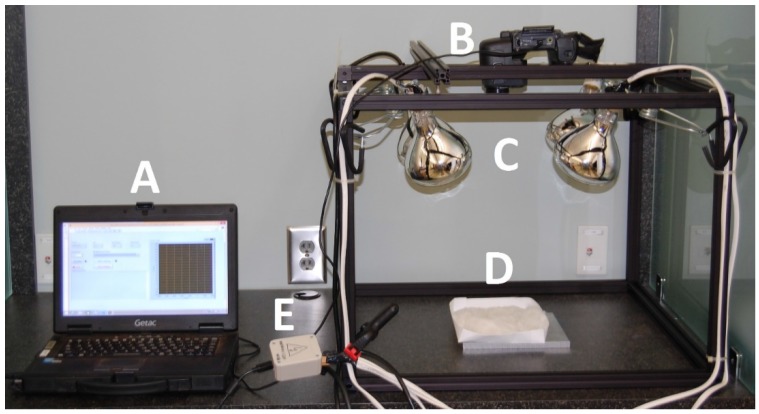
Pulsed thermography system physical components. (**A**) operating terminal; (**B**) thermal camera; (**C**) heat lamps; (**D**) sample stage; (**E**) USB power relay for lamp control.

A pulsed thermography system was constructed to facilitate the performance of pulsed thermographic analysis (Figure 3). A general-use laptop (Getac S400, Windows 8.1 Pro 64-bit, Intel Core i5-4210M CPU, 8 GB RAM, Baoshan, Hsinchu, Taiwan) with LabVIEW (National Instruments, Austin, TX, USA) installed served as the operating terminal. Videos were collected by a FLIR (FLIR Systems, Wilsonville, OR, USA) T440 thermal camera mounted on a frame of Thorlabs (Thorlabs, Newton, NJ, USA) 25 mm steel rails and oriented towards the nadir. The T440 uses a focal plane array uncooled microbolometer with a resolution of 320 × 240 pixels, a sensitivity range of 7.5 to 13 μm, and a noise-equivalent temperature difference of 0.045 °C. The dimensions of the frame were 24′′ wide by 24′′ long by 18′′ tall. Four 325-watt Sunlite (Sunlite, Brooklyn, NY, USA) heat lamps with adjustable clamp mounts provided thermal stimulation. A stainless steel plate was used as the sample stage (stainless steel is highly reflective in infrared wavelengths, minimizing heating due to the heat lamps and therefore maximizing contrast between the sample and background in collected videos). A USB-operated power relay module was used to activate and deactivate the lamps with high precision.

**Figure 4:**
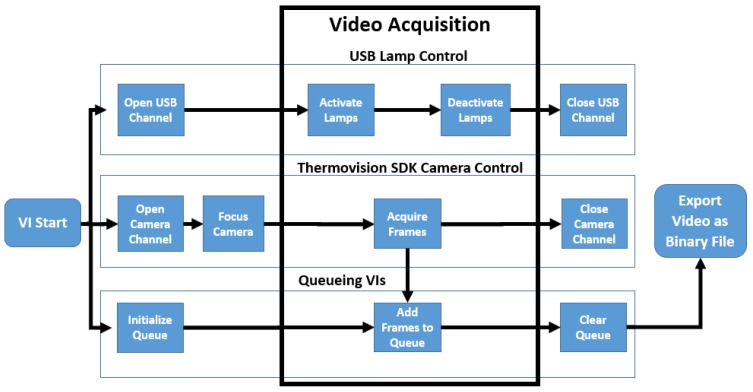
Pulsed thermography system automation virtual instrument flowchart.

**Figure 5:**
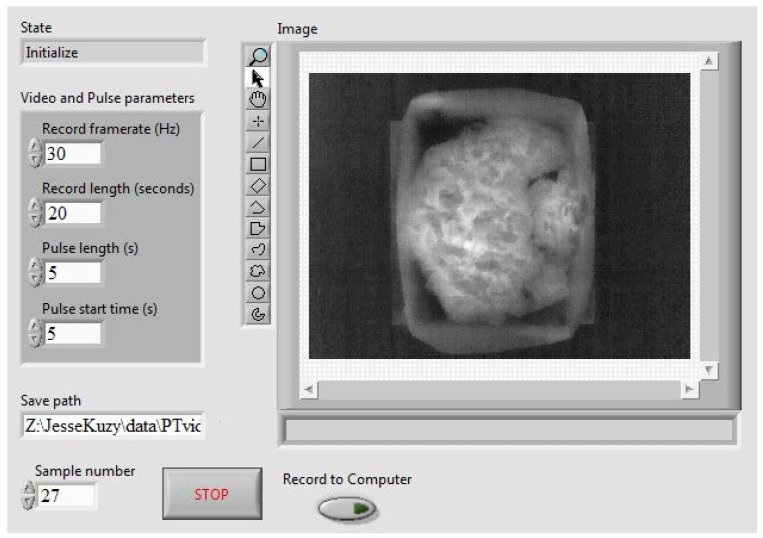
Pulsed thermography system automation virtual instrument front panel.

A LabVIEW Virtual Instrument (VI) was created to automate the operation of this system (Figure 4 and Figure 5). It consisted of three main tasks performed in parallel: activation and deactivation of the heat lamps, operation of the thermal camera, and memory management to rapidly acquire videos with no frame loss.

VIs from the FLIR ThermoVision SDK were used to operate the thermal camera and receive frames during video acquisition. To operate the USB power relay module, VIs were used which call functions from a third-party digital link library. VIs from LabVIEW’s queueing system enabled high-speed data acquisition to memory. Lastly, videos were exported as binary files; other methods of export available in LabVIEW were not possible due to the relatively large file size of collected videos (approx. 300 megabytes).

**Figure 6:**
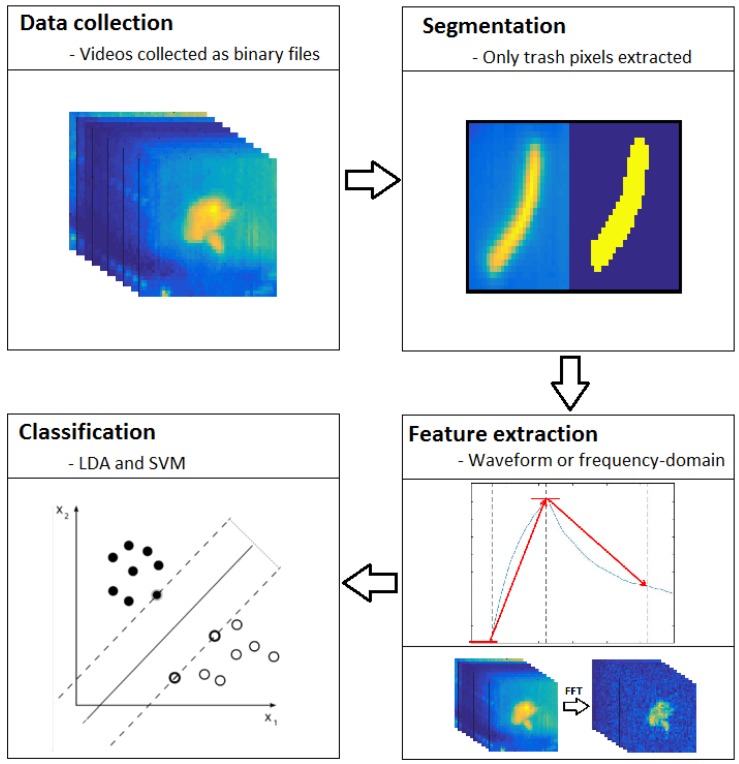
Diagram of the data processing pipeline.

Videos of pulsed thermographic analysis were collected in the following format: a front buffer of 1 s; 5 s of thermal stimulation from the heat lamps; 10 s of cooling; and a rear buffer of 1 s. Videos were collected during two sessions in May 2016. A total of 240 videos, one each for each sample, were collected. The overall data processing pipeline was performed in four main steps (Figure 6): data collection, segmentation, feature extraction, and classification.

### 2.3. Segmentation

Samples were segmented in the following fashion: for cotton samples, a 100-by-100-pixel window from the center of each video was extracted. For paper samples, a rectangular region from the center of each sample was selected, taking care to leave ample space between the selected region and the dark border marked with a permanent marker on each sample. For all other samples, Otsu thresholding of the frame of peak temperature was used to create segmentation masks. Where necessary, the threshold was manually adjusted to produce accurate masks. All portions of the segmentation procedure were performed in MATLAB (The MathWorks, Inc., Natick, MA, USA).

### 2.4. Feature Extraction

#### 2.4.1. Frequency-Domain Features

Two sets of features were extracted from the segmented videos: pulse-phase thermography features of amplitude and phase values from complex components produced by Fourier analysis, and waveform features produced by analyzing the temperature waveforms of each pixel of each sample.

**Figure 7:**
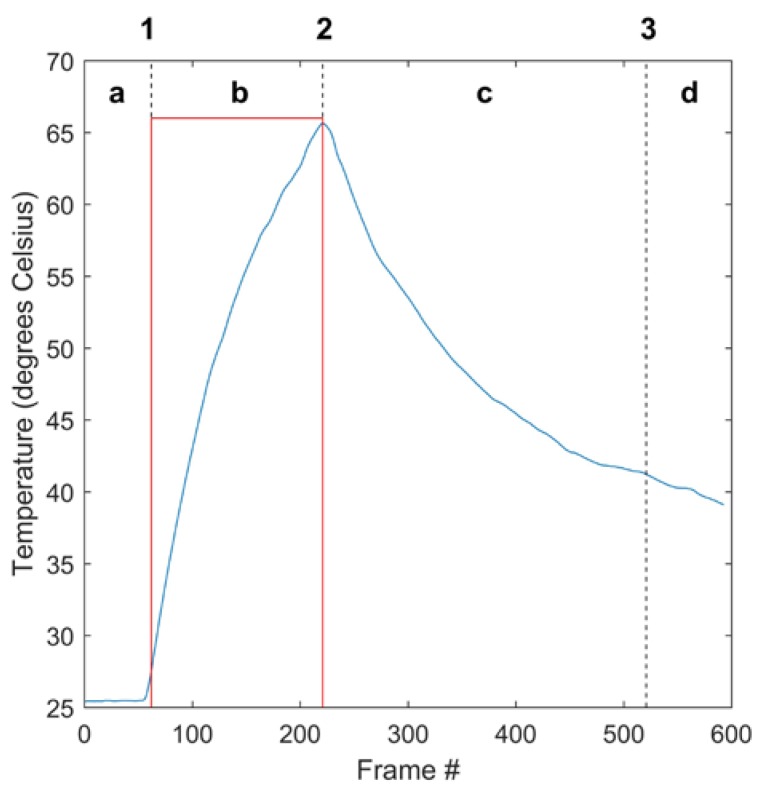
Segmented mean temperature waveform of cotton sample #4. (**a**) resting stage/front buffer; (**b**) thermal stimulation; (**c**) cooling; (**d**) rear buffer; (**1**) lamp activation; (**2**) lamp deactivation; (**3**) data cutoff. The red square wave represents the thermal stimulus provided by the heat lamps and shows the times of activation and deactivation. The magnitude of the square wave is here arbitrary.

Fourier analysis of the samples was performed using MATLAB’s fast Fourier transform (FFT) algorithm. This process decomposes the input signal into a sum of sinusoids expressed as complex phasors with evenly-spaced frequencies ranging from 0 to 30 Hz (the framerate of the acquiring device). Prior to Fourier analysis, each video was trimmed to only the rising and falling portions, with the pre- and post-stimulation buffers removed. This resulted in videos of precisely 450 frames. The input signal for Fourier analysis was the mean temperature of all of the sample’s pixels during each frame, such as that shown in Figure 7. Following Fourier decomposition, a number of complex components equal to the number of frames in each video (450) were produced. According to the Nyquist theorem, all components with a frequency higher than half of the collection frequency are aliased and therefore contain no additional information. Examination of the phase and amplitude values of the components confirmed this. Accordingly, the 225 components with frequencies higher than 15 Hz were discarded, leaving 225 components with frequencies from 0 Hz to 15 Hz. Each component, like all sinusoids, has an amplitude value and a phase value. Amplitude values were extracted by calculating the absolute value of each phasor, while phase values were determined by examining the angle of the phasor. The phase value of the 0-Hz component is always zero and was thus discarded. The final set of frequency-domain features produced by this process was 224 phase values and 225 amplitude values for each sample.

In addition to the above frequency-domain features, which were produced by analyzing the entire waveform, consisting of the rising and falling portions together, waveforms were also analyzed in a split fashion. Since many prior applications [23,28] of pulse-phase thermography examine only the falling portion of the thermal signal, it is suspected that more meaningful frequency-domain features might be produced by independently performing Fourier analysis on the rising and falling portions of the full thermal signal. Additionally, this data may be of use for implementation in ginning facilities, where a possible point of examination is immediately after the cotton exits the dryers. This was conducted, producing 223 phase values (74 rising and 149 falling) and 225 amplitude values (75 rising and 150 falling). These will be referred to in analyses as “split features”, as opposed to “whole features”.

#### 2.4.2. Waveform Features

Two waveform features were extracted from each video: peak temperature less resting temperature, and final temperature less resting temperature. The first feature was derived by subtracting the mean temperature of all of the pixels of each sample at the time labeled as 1 in Figure 7 from the mean temperature at time 2. For the second feature, the mean temperature at time 1 was subtracted from the mean temperature at time 3. These features represent the temperature gain after heating and the temperature gain after both heating and cooling, respectively.

Preliminary classification trials were performed in order to determine the optimal number of amplitude features to use. For each of the three sets of amplitude features (whole, rising, and falling), LDA and support vector machine (SVM) classifiers were trained to perform both the detection (two-class, with one class being cotton and the other foreign matter) and identification (twelve-class, with cotton lint and each foreign matter type receiving a unique class label) tasks. Cumulative sets of features ranging from the lowest-frequency component’s amplitude alone to a set consisting of the fifteen lowest-frequency amplitude values were used.

### 2.5. Statistical Analyses and Classification

In order to determine the degree of separation among the foreign matter types when all features in a given set are considered together, Hotelling’s *T*-squared tests were performed for each pair of foreign matter classes. Additionally, canonical discriminant analysis was performed on each feature set. Both tests were performed using MATLAB’s manova1 function.

Classification trials were performed in MATLAB using leave-one-out cross-validated SVM and LDA classifiers. Classification was performed using both waveform and amplitude features.

## 3. Results

### 3.1. Waveform Feature Analysis

**Figure 8:**
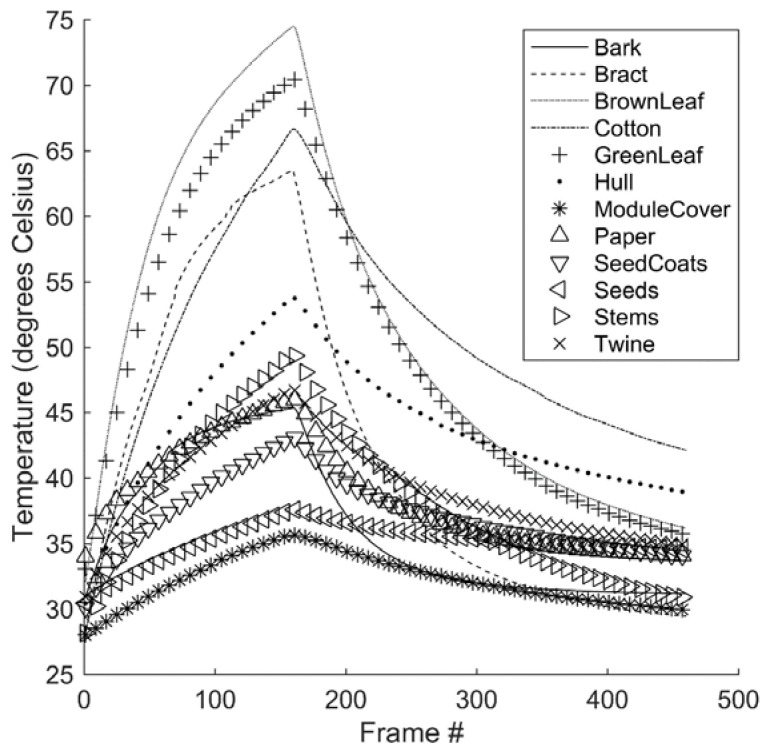
Mean thermal waveforms for all sample classes.

Examining the mean thermal waveform of each foreign matter type shows that there were clear differences in the mean thermal signals of the various foreign matter classes (Figure 8). For example, it is clear that the peak temperature of brown leaf samples, with a mean value of about 75 °C, was substantially higher than that of seed coats, with a mean peak temperature of about 35 °C. Examining other features of the waveform, it can be seen that bract and cotton samples, which achieved similar peak temperatures, had very different rates of cooling: the falling slope of the bract samples was substantially steeper than that of the cotton samples.

The maximum temperature achieved by any sample class, approximately 75 °C for brown leaf, is notable for being well below the threshold temperature at which it is considered unsafe to dry cotton, 150–175 °C. Cotton may be dried at air temperatures of up to 120 °C [29]. Since this well exceeds the maximum observed temperature of foreign matter in this study, it is reasonable to conclude that the magnitude of thermal change that results from drying will meet or exceed those observed in this study, and will therefore be sufficient to produce differences in thermal waveforms. This strengthens the possibility of implementing this technique without the need for exogenous heat sources. Conversely, this also suggests that the procedure, if conducted in isolation from the ginning process, poses no risk of overheating and damaging the cotton.

### 3.2. Frequency-Domain Feature Analysis

**Figure 9:**
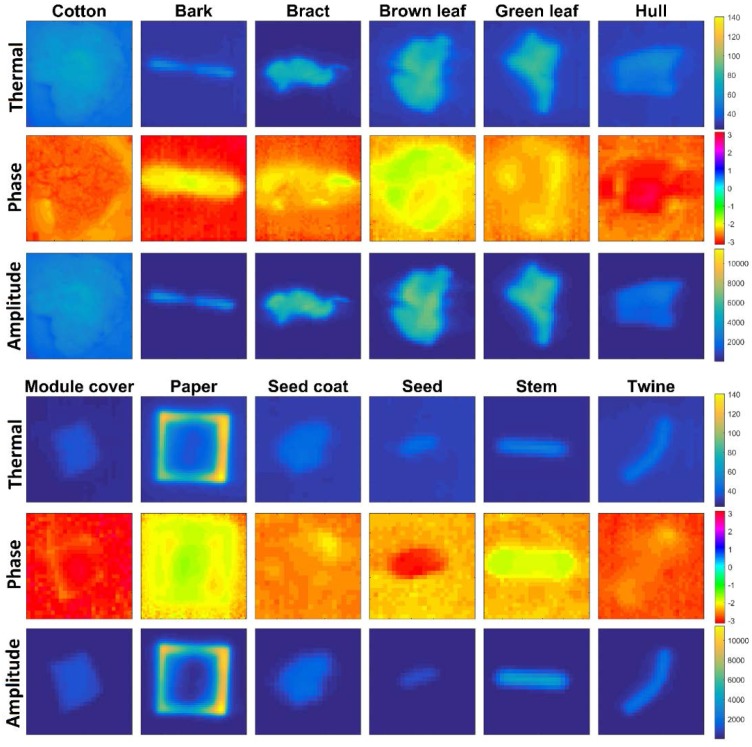
Thermal images, phasegrams, and ampligrams of each sample type.

Phase and amplitude data can be visualized by mapping the phase or amplitude values of a selected frequency component for each pixel in an image to a color map. The resulting images are known as phasegrams and ampligrams, respectively (Figure 9). In the thermal images, it can be seen that bract, brown leaves, and green leaves achieve the highest temperatures, owing to the particulars of their geometry (broad and thin); ampligrams are primarily a reflection of this peak temperature, appearing as nearly identical to the thermal images, though with de-noised backgrounds. Phasegrams are more difficult to interpret: they represent the dynamics of how quickly differing regions heat and cool. Thus, for example, the edges of the module cover sample are clearly visible, implying a difference between the rate of heating and cooling between the edges and center. Likewise, samples with a linear geometry, such as bark, stem, and twine, all show a characteristic difference in phase values between the tips and the centers.

**Figure 10:**
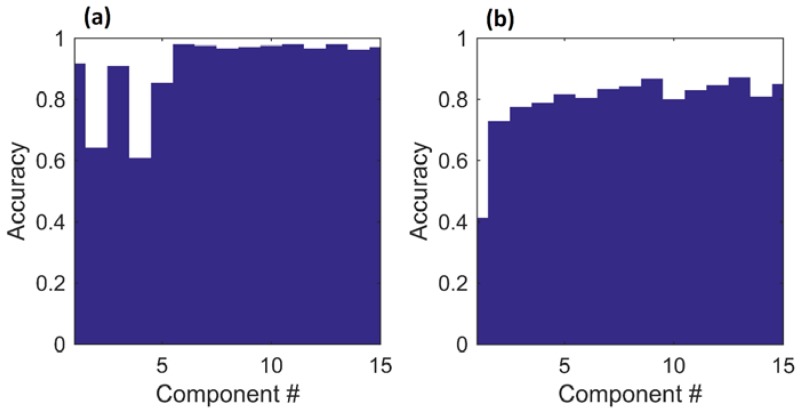
Results of preliminary amplitude feature classification trials using whole amplitude features and SVM classifiers. (**a**) two-class task; (**b**) twelve-class task.

The accuracies produced by the preliminary feature selection trials were used to determine the optimal number of amplitude features in the range from 1 to 15 components (Figure 10). For the two-class task, accuracy was unstable until at least six components are used. For the twelve-class task, accuracy rose until nine components were used, and then fluctuated. Based on these preliminary trials, the first ten amplitude features were selected for use in further classification trials and statistical analyses.

### 3.3. Statistical Analyses

**Figure 11:**
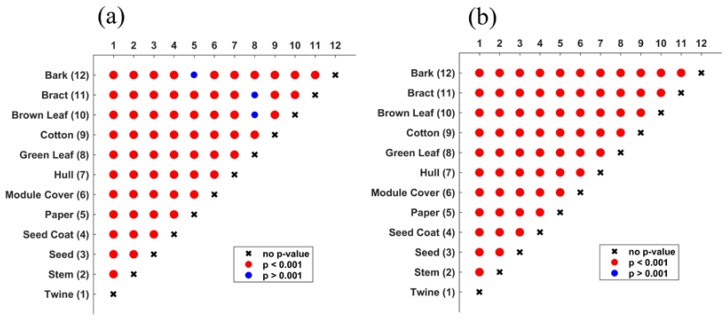
Pairwise *p*-values returned by Hotelling’s *T*-squared test performed on different feature sets. (**a**) waveform features; (**b**) amplitude features.

In the results of the paired Hotelling’s tests (Figure 11), it can be seen that, for waveform features, almost every *p*-value between two groups was well below the stringent threshold *p* = 0.001. Exceptions to this were: bark and paper, bract and green leaves, and bract and brown leaves. For amplitude features, all *p*-values were below 0.001, indicating statistically significant separation for all foreign matter types.

### 3.4. Canonical Discriminant Analysis

**Figure 12:**
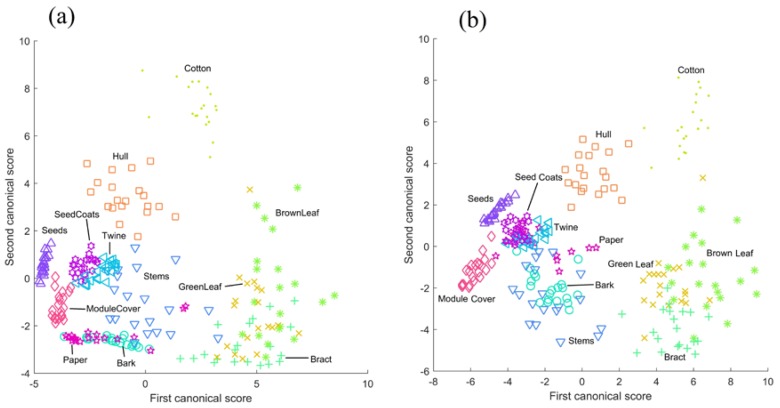
Canonical discriminant analysis scores plots from feature sets. (**a**) waveform features; (**b**) amplitude features.

The results of canonical discriminant analysis performed using the waveform features (Figure 12a) showed that there was good separation between many foreign matter types. Seeds, module cover, hull, and cotton samples were especially well-separated. Bract, brown leaves, and green leaves formed a combined cluster; given their biological and material similarity, this is unsurprising. Other foreign matter types were less well-separated: paper, stems, and bark were mixed, and twine and seed coats were only moderately well-separated.

The results of canonical discriminant analysis performed on amplitude features showed similarly good separation between many classes (Figure 12b). What is most notable about this canonical scores plot is its strong resemblance to the canonical scores plot of the waveform features. Cotton, hull, module cover and seed samples were cleanly separated and clustered; bract, green leaves, and brown leaves formed a complex; seed coats were clustered but not well-separated, and stems, bark, and paper were not well-separated. Although it is a subjective analysis, the strong similarity of the canonical score plots of the waveform and amplitude features seems to suggest that there is a large degree of overlap in the discriminating information contained by these feature sets.

### 3.5. Classification Results

The results of the classification trials produced very good results for the two-class detection problem, with more mixed results for the twelve-class identification problem (see Table 1). In the detection task, no combination of classifier and feature set produced an accuracy lower than 93%, while two trials returned accuracies above 99%. For the identification task, LDA achieved 90% accuracy of classification using whole amplitude features, while SVM provided 86.67% accuracy on whole amplitude features. For both detection and identification, the features which produced the best performance for SVM classifiers were whole amplitude features. With either classifier, whole amplitude features produced better accuracies than either rising or falling amplitude features. In general, LDA and SVM accuracies on most tasks were comparable, with the most notable exceptions being the identification task when performed using waveform or rising amplitude features.

**Figure 13:**
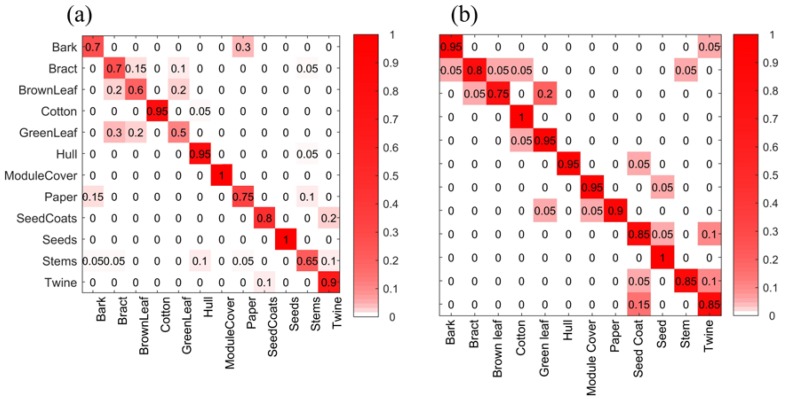
Confusion matrix for identification task performed using LDA with (**a**) waveform features and (**b**) amplitude features. *y*-axis is ground truth, *x*-axis is classifier output.

Examining the confusion matrix for the output of the identification task using LDA and waveform features (Figure 13a), it can be seen that two foreign matter types were classified with 100% accuracy, and two with better than 90% accuracy. The two worst-performing classes, green leaves and brown leaves, had most errors as confusions of one another. Considering that there is no substantial difference between these classes in their impact on the quality of the cotton lint, this confusion may be excusable. Bract was also frequently confused with both green and brown leaves, and vice versa. Since bract is physiologically and materially very similar to leaves, this is understandable. Another significant error was the classification of bark samples as paper and vice versa. Since paper is essentially homogenized, bleached wood pulp, it is unsurprising that its thermal properties are similar to thin strips of wood such as the bark samples. All of these misclassifications are in agreement with the results of the paired *t*-tests, finding no statistical significance between bark and paper or between bract and green and brown leaves, and with the canonical scores plot of waveform features, in which green leaves, brown leaves, and bract form a single complex, and paper and bark show significant overlap. It is also notable that only a single cotton sample was misclassified, and no sample was misclassified as cotton: differentiation between cotton and all foreign matter samples was performed with high accuracy.

Classification errors were more mixed for the identification task using LDA and whole amplitude features (Figure 13b). Brown leaf was the most-misclassified class, for which the most common mislabeling was green leaves, but the inverse misclassification is not present. Bract samples were also misclassified often, with a wide variety of mislabelings. Just two classes were classified with perfect accuracy: cotton and seeds; however, for this feature set, two samples were erroneously classified as cotton, which in an implemented system would amount to these foreign matter samples passing undetected. Although the overall accuracy was only marginally lower than that using waveform features, the misclassifications were more scattered and less easily explained, suggesting that amplitude features do not compare favorably to waveform features for the identification task.

These results compare well with the accuracies obtained by researchers using other methods: Fortier et al.’s classification rates of 97% and 98% using FT-NIR spectrography are superior but are based on datasets with fewer foreign matter types (four and eight, respectively), and which did not include cotton. The maximal detection accuracy of 99.58% well exceeds Yang et al.’s detection accuracy of 92% [6,7], but does not top the 100% accuracy of Zhang et al. using shortwave infrared hyperspectral imaging [13]. Although the results for bark are superior to those of Zhang et al., those for stems are not, and it should be noted that Zhang et al. considered inner and outer bark and stem surfaces as separate categories, with most misclassification for these foreign matter types being the complementary category. Fortier et al.’s best results, those using the OPUS IDENT software (Bruker Corporation, Billerica, MA, USA) with the NIR spectrum first derivative, surpass those for hulls and stems using either waveform or amplitude features, and equal those for leaves using waveform features (Fortier et al. did not consider two colors of leaves but only one category). For seed coats, their 91% accuracy exceeds the 85% achieved with amplitude features but not the 100% produced by waveform features [9].

Since cotton is dried several times during ginning, it is possible that this thermal stimulation may produce data suitable for thermographic analysis. This would eliminate the need for additional exogenous heat sources. However, two reservations should be noted: first, cotton driers function on convective heating, not radiative heating, as was used in this study. This may entail changes to the thermal responses of the cotton and foreign matter, which warrants more in-depth studies. However, cooling should remain relatively unchanged, hence the examination in this study of amplitude features drawn only from the falling portion of the thermal waveform. Second, cotton drying temperatures are frequently adjusted according to the condition of the cotton being processed at the time. This variation necessitates a system that can adjust to multiple drying temperatures. These factors need to be considered in the implementation of such a system in a ginning facility.

## 4. Conclusions

The pulsed thermographic imaging system developed by this study was proven to be effective in discriminating between cotton foreign matter types. Classification tasks using LDA and SVM classifiers produce near-perfect detection of foreign matter using both waveform and frequency domain feature sets, and respectable accuracies of identification comparable to and in some cases exceeding those achieved by other groups. Waveform features provided perfect discrimination of cotton from foreign matter types using LDA classifiers. This technique is a natural fit for the cotton processing floor, on which cotton already undergoes significant rapid heating and cooling. These findings strongly recommend pulsed thermography as a method for the detection of foreign matter in cotton lint.

## Figures and Tables

**Table 1:** Classification accuracies on two-class (detection) and twelve-class (identification) tasks using leave-one-out cross-validated LDA and SVM classifiers and four feature sets.

**Feature Set**	**Number of Features**	**Detection**		**Identification**	
		LDA	SVM	LDA	SVM
**Waveform features**	2	0.9833	0.9917	0.7375	0.7917
**Whole amp 1:10**	10	0.9917	0.9792	0.9000	0.8667
**Rising amp 1:10**	10	0.9500	0.9458	0.7292	0.7708
**Falling amp 1:10**	10	0.9625	0.9375	0.7500	0.7708
